# Rail-BEV: A LiDAR-Centric and Sensor-Aware BEV Perception Framework for Long-Range Railway Obstacle Detection

**DOI:** 10.3390/s26123637

**Published:** 2026-06-07

**Authors:** Jinghan Huang, Wentao Hu, Zifeng He, Chixiang Ma, Wenbo Song, Xinci Liu, Mingxin Yang

**Affiliations:** 1 School of Civil Engineering and Architecture, East China Jiaotong University, Nanchang 330013, China; 2023011012000117@ecjtu.edu.cn (Z.H.); 2023011012000129@ecjtu.edu.cn (W.S.); 2023011012000116@ecjtu.edu.cn (M.Y.); 2 State Key Laboratory of Safety and Resilience of Civil Engineering in Mountain Area, East China Jiaotong University, Nanchang 330013, China; walter@ecjtu.edu.cn; 3 School of Information and Software Engineering, East China Jiaotong University, Nanchang 330013, China

**Keywords:** railway obstacle detection, LiDAR-centric perception, RGB–LiDAR fusion, BEV perception, rail geometry, long-range perception, rail-aware refinement

## Abstract

Reliable long-range onboard perception is a prerequisite for future railway safety systems, where potential obstacles must be recognized under long braking distances, sparse far-field returns, and strongly constrained rail-corridor geometry. This paper presents Rail-BEV as an initial reproducible baseline study for LiDAR-centric, sensor-aware bird’s-eye-view (BEV) railway obstacle perception. LiDAR is used as the primary geometric sensing modality, while a front-center RGB camera provides lightweight auxiliary visual evidence through calibrated LiDAR-to-image projection. The aligned geometric and visual cues are organized within a unified railway-oriented BEV backend that integrates geometry-aware fusion, rail-geometry prediction, and lightweight inference-time structural refinement. Evaluation was conducted on a scene-isolated railway benchmark with range-stratified center-distance matching, and all model variants were assessed on independent test sequences rather than on validation-selected checkpoints. Compared with CenterPoint and BEVFusion baselines evaluated under the same settings, Rail-BEV achieved the highest overall mAP of 0.6669, with particularly improved long-range pedestrian perception. The controlled ablation further shows that front-view RGB evidence improves the LiDAR-only baseline from 0.5612 to 0.5750 mAP, while ROI-based rail-corridor refinement further increases mAP to 0.5916 and Rail-BEV mIoU to 0.1193. These results indicate that LiDAR-centered sensing, lightweight visual assistance, and coarse rail-aware structural reasoning can be jointly organized to support reproducible long-range railway obstacle perception. This study also clarifies the remaining limitations in rail-geometry quality, calibration robustness, sensor degradation, and strict railway-oriented localization.

## 1. Introduction

Reliable long-range onboard perception is a fundamental prerequisite for future railway safety systems. Unlike road vehicles, trains operate with significantly longer braking distances, limited maneuverability, and a tightly constrained rail corridor geometry. Consequently, potential obstacles must be detected well before they pose immediate intrusion risks. This requirement is particularly demanding in mainline railway environments, where safety-critical objects often appear at long ranges, within narrow clearance regions, and against complex trackside backgrounds. Therefore, railway obstacle perception should be treated not as a direct extension of open-road autonomous-driving detection, but as a structured sensing problem governed by railway geometry, onboard sensor topology, and application-specific evaluation metrics.

LiDAR is well suited for railway perception. Its active metric ranging and ability to map 3D structure are key advantages [1,2,3]. However, long-range LiDAR sensing is intrinsically challenging. As distance increases, effective point density degrades. Far-field targets become sparse and fragmented in returns. In railway scenes, this sparsity is made worse by persistent structural clutter. Examples include catenary poles, signal equipment, vegetation, platform edges, and elongated trackside infrastructure. Small but safety-critical objects near the rail corridor may have weaker geometric signatures than visually prominent but irrelevant background structures. This imbalance between geometric salience and operational relevance is a core challenge for long-range railway obstacle perception [4].

Most existing 3D object detection frameworks have been developed for automotive road scenes, where object localization is typically performed over a broad, relatively isotropic bird’s-eye-view (BEV) space. Although voxel-based, pillar-based, and center-based LiDAR detectors have established strong baselines for outdoor 3D perception [5,6,7,8], their underlying spatial assumptions do not fully align with the constraints of railway sensing. In railway environments, the operational significance of a detected object is strictly conditioned by its spatial relationship to the rail corridor and the clearance envelope. An object located within the rail corridor carries fundamentally different safety implications than a similar target outside it. Thus, uniformly processing the entire BEV plane risks diluting rail-relevant features and increasing vulnerability to off-corridor clutter.

A unified BEV representation provides a natural spatial framework for multi-sensor railway perception, as it maps geometric structure, visual cues, rail geometry, and object locations into a common coordinate system [9,10]. For railway scenes, this property is invaluable: the BEV plane aligns seamlessly with the track-oriented operating space, thereby facilitating corridor-aware reasoning. However, generic BEV fusion alone is insufficient. A dedicated railway perception framework must also encode rail geometry, account for operational domain variations, and distinguish standard reproducible detection benchmarks from stricter, railway-specific localization diagnostics. This distinction is vital because overlap-based 3D metrics can become unstable for small, distant objects, whereas strict rail-corridor localization remains exceptionally challenging under sparse far-field returns [4,11].

The organization of sensor data is equally critical. Modern railway datasets provide calibrated and synchronized onboard sensing streams, enabling the investigation of multimodal perception under realistic operating conditions [12]. However, directly deploying heavy surround-view fusion architectures designed for road vehicles is sub-optimal for railway applications. The forward rail corridor represents the dominant safety-critical field of view, where LiDAR remains the most reliable source of metric geometry. A more effective strategy is to maintain LiDAR as the primary sensing modality while utilizing a front-center RGB camera to provide lightweight, auxiliary visual cues [13,14,15,16]. In this configuration, calibrated LiDAR-to-image projection serves as the geometric interface between modalities, allowing front-view visual features to be explicitly associated with LiDAR structures prior to BEV projection.

To address these challenges, this paper presents Rail-BEV, a LiDAR-centric, sensor-aware BEV perception framework for long-range railway obstacle detection. The framework adopts a forward-facing sensing configuration in which LiDAR provides the primary geometric representation, and a single front-center RGB camera supplies aligned auxiliary visual features via calibrated LiDAR-to-image projection. The aligned geometric and visual cues are integrated within a railway-oriented BEV backend that combines geometry-aware fusion, rail-geometry prediction, and lightweight inference-time structural refinement. Rather than treating Rail-BEV as a generic open-road detector, this study frames it as a dedicated railway sensing pipeline that jointly optimizes onboard sensor configurations, cross-modal geometric alignment, rail-corridor topology, operational domain behaviors, and protocol-aware evaluation metrics.

The main contributions of this study are summarized as follows:

First, Rail-BEV is formulated as a LiDAR-centric and sensor-aware railway perception framework. Instead of adopting a heavy surround-view fusion design, the proposed framework preserves LiDAR as the dominant geometric modality and uses only the front-center RGB camera as lightweight auxiliary visual evidence. This asymmetric sensing hierarchy reflects the forward-looking nature of railway safety perception and the central role of metric geometry in long-range obstacle recognition.

Second, calibrated LiDAR-to-image projection and geometry-aware BEV fusion are incorporated to associate front-view visual cues with LiDAR structure. The resulting representation organizes sparse geometric evidence and dense visual information in a unified railway-oriented BEV space, allowing visual assistance to complement far-range point-cloud responses without weakening the LiDAR-centered sensing hierarchy.

Third, a rail-geometry branch and inference-time rail-aware refinement are introduced to encode corridor-level railway structure. The rail-geometry branch is not designed as a standalone high-fidelity rail segmentation module; rather, it provides a coarse structural prior that encourages the BEV representation to preserve the spatial relationship between candidate obstacles and the rail corridor.

Finally, the experimental evaluation is reorganized under a unified sequence-level benchmark with fixed data partitions, identical training settings, and independent test-set assessment. The revised ablation study isolates the effects of front-view RGB assistance, ROI-based rail-corridor refinement, and track-consistency refinement without relying on validation-set model selection. Representative comparisons with LiDAR-only and multimodal BEV baselines are further included to place Rail-BEV within a reproducible railway perception benchmark.

## 2. Related Work

### 2.1. LiDAR-Centric 3D Perception for Structured Railway Scenes

LiDAR-based 3D perception has become a central paradigm in outdoor autonomous sensing because it provides direct metric geometry and physically interpretable spatial structure. Voxel-based and pillar-style detectors convert unordered point clouds into structured BEV representations, while center-based detectors further formulate 3D object detection as center localization and attribute regression [5,6,7,17]. These approaches have established strong baselines for autonomous-driving perception and demonstrate the effectiveness of LiDAR-centered geometry for large-scale outdoor scenes [8,18]. Their success also explains why LiDAR is particularly attractive for railway perception, where long-range geometric awareness is essential for early obstacle recognition.

Recent railway-oriented LiDAR studies have started to adapt 3D detection architectures to railway foreign-object detection. For example, Rail-PillarNet improves a pillar-based LiDAR detector for railway foreign-object perception and demonstrates that task-specific adaptation is necessary when moving from road scenes to railway scenarios [19]. In addition to learning-based detectors, dedicated LiDAR systems and rail-surface obstacle extraction algorithms have also been developed for railway safety monitoring [1,2,3,20]. Nevertheless, most existing railway LiDAR detectors still focus primarily on object-level recognition. They do not fully integrate rail-corridor geometry as a structural component of the sensing pipeline.

### 2.2. RGB–LiDAR Fusion and BEV Representation

RGB–LiDAR fusion has been widely studied because cameras and LiDAR provide complementary sensing information. LiDAR offers accurate depth and sparse geometric structure, whereas RGB images provide dense appearance and semantic cues [10,13,14,15,16,21]. Early fusion strategies often associate image features with LiDAR points or project sparse point-cloud evidence into the image plane. Although these strategies can establish local cross-modal correspondence, they may not fully exploit the dense semantic information of images or provide a consistent spatial frame for downstream detection.

Unified BEV representation has become an important direction for multi-sensor perception because it allows geometric and visual evidence to be represented in a common coordinate system [9,10,22]. BEVFusion shows that multimodal features can be organized in a shared BEV space, preserving both geometric and semantic information while supporting downstream perception tasks [16]. This idea is especially relevant to railway scenarios because the BEV plane naturally matches the track-aligned operating space. Rail geometry, obstacle positions, and corridor constraints can be interpreted more coherently in BEV than in independent sensor coordinate systems.

For railway applications, however, sensor fusion must be designed according to the sensing hierarchy of the platform, rather than directly importing heavy surround-view camera architectures from road-scene autonomous driving. OSDaR23 provides calibrated and synchronized railway sensor data, including front-mounted visual sensors and LiDAR, enabling the study of multimodal railway perception under realistic onboard sensing conditions [23]. In the present work, LiDAR is retained as the primary geometric modality, while only the front-center RGB camera is used as lightweight auxiliary visual evidence [13,14]. Calibrated LiDAR-to-image projection provides the geometric interface between these modalities, allowing front-view visual cues to be associated with LiDAR structure before being organized in the BEV space. This formulation is more consistent with forward-looking railway perception than a full multi-camera fusion strategy.

### 2.3. Rail Geometry Modeling and Structural Guidance

Railway scenes are strongly structured by physical track geometry. Unlike general road environments, where objects can appear across a broad traffic space, railway obstacle relevance is tightly coupled to the rail corridor and clearance envelope. An object located inside or near the rail corridor has a different operational meaning from a visually similar object outside the corridor. This makes rail geometry an important source of structural guidance for perception [24,25].

Prior railway obstacle-detection systems have explored visual monitoring, laser-based sensing, multi-sensor onboard detection, and railway-specific 3D perception [12,26,27]. These works demonstrate that railway perception benefits from sensor configurations and spatial priors tailored to the rail environment. However, many existing strategies rely on heuristic region restriction, local filtering, or task-specific processing outside a unified BEV learning framework. Such designs can reduce background interference, but they may be difficult to generalize to curved tracks, platforms, turnouts, and complex station scenes [24,25].

A more integrated solution is to predict rail-related geometry within the perception network and use it as structural guidance for BEV-based obstacle perception. In Rail-BEV, the rail-geometry branch is therefore not treated merely as a visualization component. It provides an auxiliary structural signal that encourages the BEV representation to encode the rail corridor together with object-level evidence [24,25]. The subsequent lightweight structural refinement further uses this railway-consistent geometry to improve practical detection behavior. This design links rail geometry, LiDAR-centered sensing, and BEV obstacle perception within one pipeline.

### 2.4. Evaluation Protocols for Long-Range Railway Obstacle Perception

Evaluation is a critical issue in long-range railway perception. Conventional 3D object detection benchmarks often rely on overlap-based criteria such as 3D IoU and center-based metrics [7,18]. These metrics are useful for general object detection, but they can become unstable for small and distant targets. When an object is represented by only a few sparse LiDAR returns, small localization deviations may cause the predicted and ground-truth boxes to have little or no volumetric overlap, even if the prediction still carries warning value for railway safety [4,11].

Recent studies on 3D perception have pointed out that localization errors should be interpreted carefully under long-range and small-object conditions [4,11]. This issue is particularly important in railway applications because longitudinal and lateral errors do not have the same operational meaning. Longitudinal deviations may be influenced by range sparsity and braking-margin interpretation, whereas lateral deviations are directly related to whether an object intrudes into the rail clearance region. Therefore, a railway-oriented evaluation framework should separate the main reproducible detection benchmark from stricter safety-oriented localization diagnostics.

Following this principle, the present study reports the main benchmark using range-stratified center-distance AP/mAP together with rail BEV mIoU under a scene-isolated OSDaR23 evaluation setting [12]. The training, validation, and test partitions are separated at the scene/sequence level to reduce temporal leakage and to distinguish model development from independent evaluation. RA-AP and RA-mAP are retained as supplementary railway-oriented localization diagnostics rather than being promoted as the primary headline metric [11]. This separation is important for scientific clarity: center-distance mAP and rail BEV mIoU describe reproducible benchmark behavior, whereas RA diagnostics expose stricter localization bottlenecks under railway safety semantics.

## 3. Methodology

### 3.1. Sensor Configuration and Overall Framework

Rail-BEV is formulated as a LiDAR-centric and sensor-aware bird’s-eye-view (BEV) perception framework for long-range railway obstacle detection. The framework follows a frontal-clean onboard sensing configuration, in which LiDAR is retained as the primary geometric sensing modality and only the front-center RGB camera is used as lightweight auxiliary visual evidence. This design reflects the forward-looking nature of railway safety perception: the most safety-critical region is concentrated along the rail corridor, while LiDAR remains the most reliable source of metric geometry for long-range spatial reasoning [12,23,26,27]. The detailed sensor layout and configuration parameters are provided in Figure S1 and Table S1 of the Supplementary Materials.

**Figure 1:**
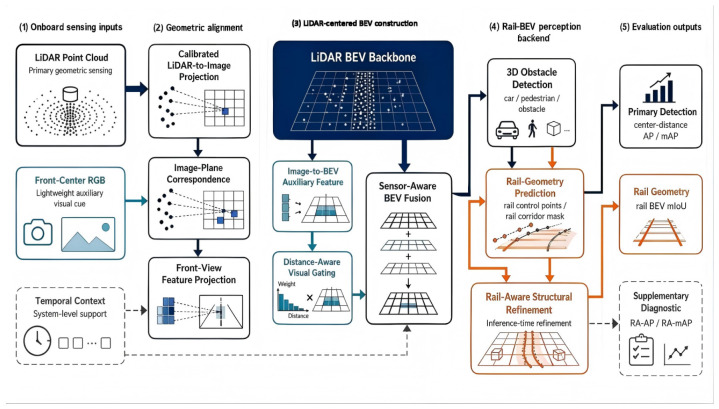
LiDAR-centric onboard sensing configuration and sensor-aware BEV perception pipeline of Rail-BEV. The arrows indicate the direction of data flow.

**Figure 2:**
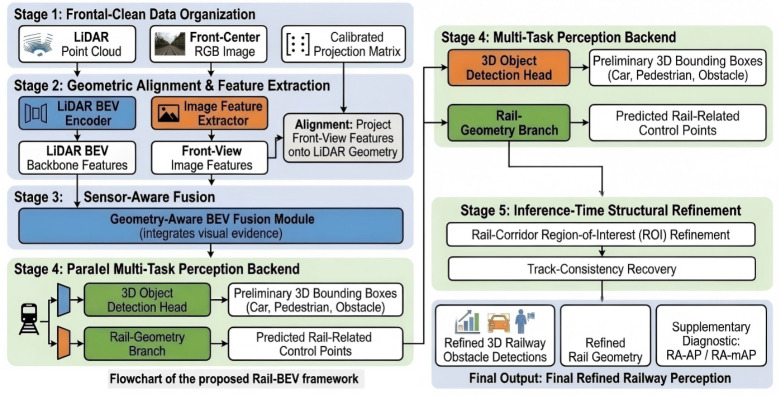
Detailed stage-wise flowchart of the proposed Rail-BEV framework. The pipeline illustrates the complete data flow from frontal-clean sensor data organization (Stage 1), geometric alignment and feature extraction (Stage 2), and geometry-aware BEV fusion (Stage 3), to the parallel multi-task perception backend (Stage 4) and inference-time structural refinement (Stage 5) for final railway obstacle perception. The arrows indicate the direction of the data flow and processing stages.

As illustrated in Figure 1, Rail-BEV organizes heterogeneous onboard sensing signals into a unified railway-oriented BEV pipeline. The LiDAR stream first establishes the dominant BEV geometric representation. The front-center RGB stream provides auxiliary appearance evidence, which is associated with LiDAR geometry through calibrated LiDAR-to-image projection. The aligned visual and geometric cues are then integrated by a geometry-aware BEV fusion module. The fused representation is forwarded to two parallel task branches: a 3D object-detection head for railway obstacle perception, and a rail-geometry branch for structural corridor modeling. Finally, lightweight inference-time structural refinement is applied to improve the practical use of rail-consistent spatial information. The detailed stage-wise data flow of the framework is presented in Figure 2.

The proposed framework is therefore not intended as a heavy multimodal detector based on full surround-view camera fusion. Instead, it is designed as a LiDAR-dominant railway sensing backend in which front-view visual evidence, rail geometry, and BEV reasoning are coupled under a compact and deployment-oriented sensing hierarchy [9,13,14].

### 3.2. Frontal-Clean Sensor Data Organization

To stabilize the sensor interface for railway perception, Rail-BEV adopts a frontal-clean data organization strategy. Instead of using all available image streams, the data pipeline retains only the front-center RGB image and pairs it with the corresponding LiDAR frame and calibrated LiDAR-to-image projection matrix. This organization reduces cross-view fusion complexity while preserving the forward visual evidence that is most relevant to railway obstacle perception [23].

For each time step t, the sensor input is represented as a triplet consisting of the LiDAR point cloud, the front-center RGB image, and the calibrated projection matrix:

(1) $$X_{t}=\left\{P_{t},I_{t},\Pi_{t}\right\},$$

where $P_{t}$ denotes the current LiDAR point cloud, $I_{t}$ denotes the aligned front-center image, and $\Pi_{t}$ denotes the LiDAR-to-image projection matrix. For a LiDAR point represented in homogeneous coordinates as $p={\left[x,y,z,1\right]}^{⊤}$, its image-plane correspondence is obtained by

(2) $$\tilde{u}=\Pi_{t}p,\;u=\frac{\tilde{u}}{\tilde{w}},\;v=\frac{\tilde{v}}{\tilde{w}}.$$

### 3.3. LiDAR-Centric BEV Perception Backbone

The perception backbone follows a LiDAR-centered BEV design. The LiDAR branch converts the point cloud into a structured BEV feature representation through voxel- or pillar-style encoding and BEV feature extraction [5,6]. This branch provides the primary geometric representation for downstream railway perception, because LiDAR directly preserves metric depth and spatial structure even when visual appearance is ambiguous. The LiDAR BEV feature at time *t* is expressed as

(3) $$F_{t}^{L}=\Phi_{L}\left(P_{t}\right),$$

where $\Phi_{L}\left(\cdot \right)$ denotes the LiDAR BEV encoder and $F_{t}^{L}$ is the resulting BEV feature tensor. This representation serves as the dominant feature space of the framework. The detection and rail-geometry heads operate after sensor-aware fusion, but the geometric backbone remains LiDAR-centered throughout the pipeline.

### 3.4. Geometry-Aware BEV Fusion

The front-center RGB camera is used to provide auxiliary visual cues, rather than to replace LiDAR geometry. The image branch extracts features from the front-view image, and the calibrated projection matrix is used to associate image-space information with LiDAR geometry [9,13,14,15,16]. This design introduces visual evidence into the BEV domain while maintaining a physically interpretable sensing hierarchy.

The front-view image feature is defined as

(4) $$F_{t}^{I}=\Phi_{I}\left(I_{t}\right),$$

where $\Phi_{I}\left(\cdot \right)$ denotes the image feature extractor. The geometry-aware BEV fusion process is then formulated as

(5) $$F_{t}^{B}=G\left(F_{t}^{L},F_{t}^{I},\Pi_{t}\right),$$

where G(·) denotes the fusion operator that incorporates image evidence into the LiDAR-centered BEV representation using the calibrated projection relationship. The fused representation $F_{t}^{B}$ preserves the geometric dominance of LiDAR while allowing front-view visual cues to complement sparse far-field point-cloud evidence. This formulation follows the frontal-clean sensing design: the system benefits from lightweight camera assistance without introducing the complexity of full multi-camera BEV fusion.

### 3.5. Rail-Geometry Branch as a Coarse Structural Prior

Railway obstacle relevance is strongly coupled with rail-corridor geometry. To encode this structure, Rail-BEV introduces a rail-geometry branch that predicts rail-related control points from BEV features. The branch acts as an auxiliary structural head and is optimized jointly with the object-detection branch. Its role is not to provide high-fidelity dense rail segmentation but to supply a coarse corridor-level structural prior that encourages the shared BEV representation to preserve rail-related spatial organization useful for railway obstacle perception [24,25].

Let the predicted and reference rail-control-point sets be defined as

(6) $${\hat{R}}_{t}={\left\{r_{i}\right\}}_{i=1}^{M},\;R_{t}={\left\{r_{j}\right\}}_{j=1}^{K}.$$

The geometric discrepancy between the predicted and reference rail structures is measured by the Chamfer distance [28]:

(7) $$d_{\mathrm{CD}}\left({\hat{R}}_{t},R_{t}\right)=\frac{1}{M}\sum_{i=1}^{M}\min_{r_{j}\in R_{t}}∥{\hat{r}}_{i}-r_{j}{∥}_{2}^{2}+\frac{1}{K}\sum_{j=1}^{K}\min_{{\hat{r}}_{i}\in {\hat{R}}_{t}}{∥r_{j}-{\hat{r}}_{i}∥}_{2}^{2}.$$

The overall training objective combines the detection loss and the rail-geometry loss:

(8) $$L_{\mathrm{total}}=L_{\det}+\lambda_{\mathrm{rail}}L_{\mathrm{rail}}.$$

where $L_{\det}$ denotes the object-detection loss, $L_{\mathrm{rail}}$ denotes the rail-geometry loss derived from the predicted rail structure, and $\lambda_{\mathrm{rail}}$ controls the contribution of the structural branch. This formulation keeps the rail branch as an auxiliary geometric constraint and avoids reducing the detection objective to a single bounding-box regression term.

### 3.6. Inference-Time Structural Refinement

In addition to the neural BEV backend, Rail-BEV uses lightweight inference-time structural refinement to improve the practical use of railway-consistent spatial information. The refinement stage operates after raw predictions are generated and uses the predicted rail geometry to adjust detection behavior in a structurally informed manner. Its purpose is to suppress rail-inconsistent responses and recover rail-consistent candidates without imposing a fixed universal confidence-decay rule.

Let $y_{t}^{\mathrm{base}}$ denote the raw detection output from the BEV detector, and let ${\hat{R}}_{t}$ denote the predicted rail structure. The refined output is formulated as

(9) $$y_{t}^{*}=H_{D}\left(H_{C}\left(y_{t}^{\mathrm{base}},{\hat{R}}_{t}\right)\right),$$

where $H_{C}\left(\cdot \right)$ denotes rail-corridor geometry refinement and $H_{D}\left(\cdot \right)$ denotes track-consistency recovery. This composition describes structural refinement at the system level. It replaces the older fixed off-rail confidence-penalty formulation and avoids treating rail consistency as a hard binary mask. Under the revised independent-test evaluation, ROI-based rail-corridor refinement is interpreted as the main stable structural component, whereas the additional track-consistency step is treated as a supplementary refinement variant with limited incremental benefit in the current experiments.

### 3.7. Offline Class-Balanced Resampling

Railway obstacle datasets often exhibit long-tailed category distributions. Frequent or background-associated categories may dominate the optimization process, while rare but safety-critical obstacle instances remain underrepresented. To reduce this imbalance without increasing inference-time complexity, Rail-BEV adopts offline class-balanced resampling during data preparation.

Let $N_{c}$ denote the number of training samples associated with category *c*. The reweighted sampling probability is defined as

(10) $$p^{\prime }\left(c\right)\propto \frac{1}{N_{c}^{\alpha }},\;\alpha \geq 0,$$

where α controls the strength of class rebalancing. The corresponding learning objective can be expressed as

(11) $$J\left(\theta \right)=E_{\left(x,y\right)∼p^{\prime }}\left[L_{\mathrm{total}}\left(f_{\theta }\left(x\right),y\right)\right].$$

Because this rebalancing is performed before training, it does not introduce additional computational cost during inference. In the present framework, class-balanced resampling is treated as a supporting training strategy rather than as the sole driver of the final benchmark gain.

### 3.8. Stage-Wise Training Strategy

Rail-BEV adopts a stage-wise training strategy to improve optimization stability and separate different aspects of the sensing pipeline. The first stage establishes a geometry-reliable LiDAR backbone. The second stage adapts the LiDAR-centered detector to real railway sensing conditions. The third stage introduces the frontal-clean visual configuration and fine-tunes the sensor-aware BEV fusion pipeline using aligned LiDAR-RGB assets.

This process can be expressed as

(12) $$\theta^{\left(1\right)}=\arg\min_{\theta }L^{\left(1\right)}\left(\theta \right),\;\theta^{\left(2\right)}=\arg\min_{\theta }L^{\left(2\right)}\left(\theta ;\theta^{\left(1\right)}\right),\;\theta^{\left(3\right)}=\arg\min_{\theta }L^{\left(3\right)}\left(\theta ;\theta^{\left(2\right)}\right).$$

Here, $\theta^{\left(1\right)}$, $\theta^{\left(2\right)}$, and $\theta^{\left(3\right)}$ denote the parameters obtained after LiDAR-centered pretraining, real-domain adaptation, and sensor-aware fine-tuning, respectively. This staged design follows the sensing hierarchy of the framework: stable LiDAR geometry is learned first, and lightweight visual assistance is introduced only after the core BEV detector has become sufficiently reliable.

### 3.9. Main Benchmark and Supplementary Diagnostics

The main benchmark of Rail-BEV is based on range-stratified center-distance AP/mAP together with rail BEV mIoU. This choice is motivated by the difficulty of evaluating small and distant railway objects using strict overlap-based criteria [11,29]. A prediction is matched to a reference object when the Euclidean center-distance criterion is satisfied:

(13) $$∥\hat{c}{-c∥}_{2}\leq \tau_{c},$$

where $\hat{c}$ and c denote the predicted and reference object centers, respectively, and $\tau_{c}$ is the class-specific center-distance threshold.

Rail-geometry consistency is evaluated by rasterizing predicted and reference rail structures into BEV occupancy masks. The rail BEV mIoU is calculated as follows:

(14) $$\mathrm{Rail}\_\mathrm{BEV}\_\mathrm{mIoU}=\frac{|\hat{M}\cap M|}{|\hat{M}\cup M|+\varepsilon },$$

where $\hat{M}$ and *M* denote the predicted and reference BEV rail masks, and ε is a small constant used for numerical stability.

In addition to the main benchmark, RA-AP and RA-mAP are retained as supplementary railway-oriented localization diagnostics. These diagnostics are not used as the primary headline metric, but they help expose the remaining difficulty of strict rail-corridor localization [11]. For a predicted box and a reference box, the decoupled elliptical localization distance is defined as follows:

(15) $$d_{\mathrm{elli}}\left(B_{\mathrm{pred}},B_{\mathrm{gt}}\right)=\sqrt{{\left(\frac{\Delta_{\|}}{a_{c}}\right)}^{2}+{\left(\frac{\Delta_{⊥}}{b_{c}}\right)}^{2}}.$$

Here, $\Delta_{\|}$ and $\Delta_{⊥}$ denote the longitudinal and lateral center deviations in the rail-oriented coordinate frame, while $a_{c}$ and $b_{c}$ denote class-specific longitudinal and lateral tolerances. A prediction is considered matched under this diagnostic rule when

(16) $${\left(\frac{\Delta_{\|}}{a_{c}}\right)}^{2}+{\left(\frac{\Delta_{⊥}}{b_{c}}\right)}^{2}\leq 1.$$

This evaluation design separates reproducible center-distance detection performance from stricter railway-oriented localization analysis. Consequently, center-distance AP/mAP and rail BEV mIoU are treated as the main benchmark metrics, while RA-AP and RA-mAP are interpreted only as supplementary diagnostics for safety-relevant localization behavior.

## 4. Results

This section reports the reproducible performance of Rail-BEV under a scene-isolated OSDaR23 evaluation setting [12,23]. The results are organized to separate representative baseline comparison, controlled ablation analysis, operating-domain diagnostics, and supplementary railway-oriented localization analysis. Center-distance AP/mAP and rail BEV mIoU are treated as the primary metrics, whereas RA-AP and RA-mAP are retained only as supplementary railway-oriented localization diagnostics.

### 4.1. Experimental Protocol

The benchmark was conducted on public OSDaR23 sequences using a fixed scene-level split. Instead of random frame-level sampling, the data were partitioned at complete sequence boundaries to reduce temporal correlation between training and evaluation frames. The revised benchmark contains 23 training sequences with 583 frames, 5 validation sequences with 50 frames reserved for internal hyperparameter verification, and 5 independent test sequences with 230 frames for final reporting. LiDAR was retained as the primary geometric sensing modality, while only the front-center RGB camera was preserved as the auxiliary visual sensor. This configuration maintains a LiDAR-centric sensing hierarchy while retaining the most safety-relevant forward-view visual evidence for calibrated RGB–LiDAR association.

The primary detection metric was range-stratified center-distance AP/mAP, which was used as the headline benchmark for obstacle detection under sparse long-range railway sensing. Rail-geometry consistency was evaluated using rail BEV mIoU, which served as a companion metric for assessing whether the learned rail structure remained consistent in the BEV space. In addition, RA-AP and RA-mAP were reported separately as supplementary railway-oriented localization diagnostics. These diagnostic metrics were not used as the primary benchmark headline; rather, they were retained to expose strict rail-corridor localization bottlenecks that may not be fully reflected by center-distance matching alone.

### 4.2. Baseline Realignment and Distance-Stratified Evaluation

**Figure 3:**
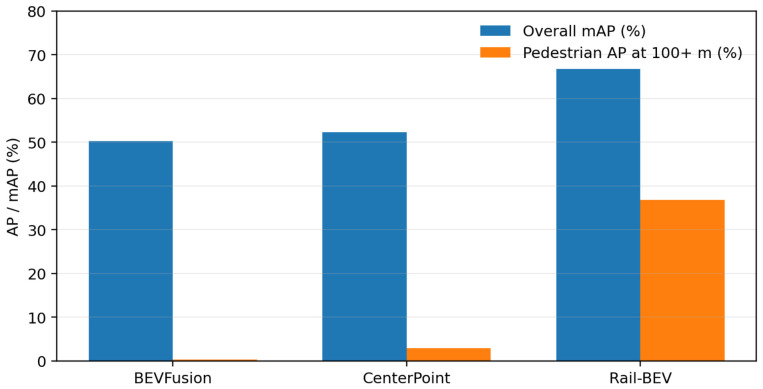
Distance-stratified baseline comparison under the scene-isolated OSDaR23 evaluation setting. The figure summarizes the overall mAP and the ultra-long-range pedestrian AP of BEVFusion, CenterPoint, and Rail-BEV, highlighting the long-range degradation behavior of competing baselines.

Table 1 compares Rail-BEV with representative CenterPoint and BEV Fusion baselines under the same scene-isolated evaluation setting. The comparison is reported across three physical distance bands: 0–50 m, 50–100 m, and 100+ m. This range-stratified design translates the numerical AP values into a more interpretable physical performance profile, addressing the fact that object size, LiDAR point density, and localization uncertainty change substantially with distance, see Figure 3.

Rail-BEV achieves an overall mAP of 66.69%, outperforming CenterPoint by 14.45 percentage points and BEVFusion by 16.51 percentage points under the aligned setting. The improvement is most evident for pedestrians at long range, where Rail-BEV maintains 36.81% AP in the 100+ m band, compared with 2.86% for CenterPoint and 0.34% for BEVFusion. This result suggests that railway-oriented structural reasoning helps constrain the search space along the rail corridor when far-field LiDAR returns become sparse.

### 4.3. Clean Ablation Under the Unified Evaluation Setting

Table 2 presents the controlled ablation results obtained under the same scene-isolated evaluation setting. All variants were trained using identical optimization settings, random seed, epoch budget, sensor configuration, checkpoint rule, and evaluation metrics. Validation-set checkpoint selection was not used; each variant was evaluated once on the independent test sequences using the final training checkpoint. The LiDAR-only baseline achieved 0.5612 mAP and 0.0533 rail BEV mIoU.

**Figure 4:**
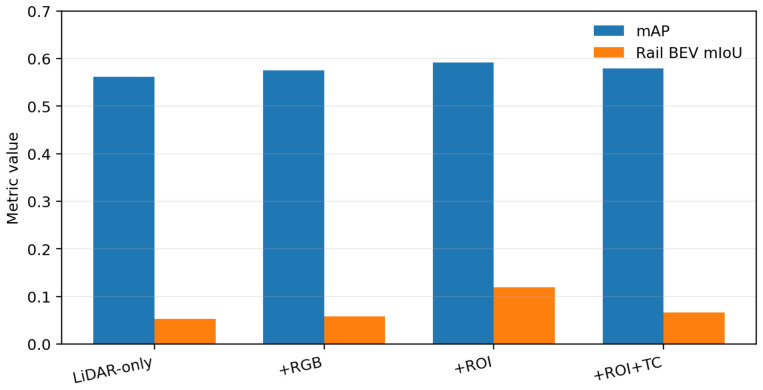
Controlled ablation analysis of Rail-BEV. ROI-based rail-corridor refinement provides the most stable improvement in overall mAP and rail BEV mIoU, whereas the additional track-consistency refinement provides limited incremental gain under the current fixed evaluation setting.

Adding the front-center RGB stream improved mAP to 0.5750 and increased the obstacle AP from 0.2270 to 0.3398, indicating that lightweight visual evidence can complement sparse LiDAR responses. ROI-based rail-corridor refinement further improved mAP to 0.5916 and increased the rail BEV mIoU to 0.1193. In contrast, the additional track-consistency refinement reached 0.5793 mAP and 0.0665 rail BEV mIoU, showing limited incremental benefit under the current fixed protocol, see Figure 4.

### 4.4. Evaluation Scope and Operating-Domain Diagnostics

Table 3 summarizes the scene-level data partition used in the revised evaluation. The benchmark is intentionally split at the sequence boundary rather than at the frame level, so that temporally adjacent frames from the same scene are not shared between training and testing. This design provides a stronger test of cross-scene reproducibility than the previous small validation subset, although it should still be interpreted as a controlled public-sequence benchmark rather than a complete validation of all railway operating domains.

Additional diagnostic slices are used only to interpret operating-domain variation and are not treated as replacements for the independent test benchmark. In particular, detection AP and rail BEV mIoU may not improve monotonically across all slices, because center-distance matching and rail-mask overlap quantify different aspects of railway perception. Center-distance AP reflects approximate object localization under sparse long-range returns, whereas rail BEV mIoU reflects the consistency of the predicted rail structure in the BEV space.

### 4.5. Qualitative Multimodal Evidence

**Figure 5:**
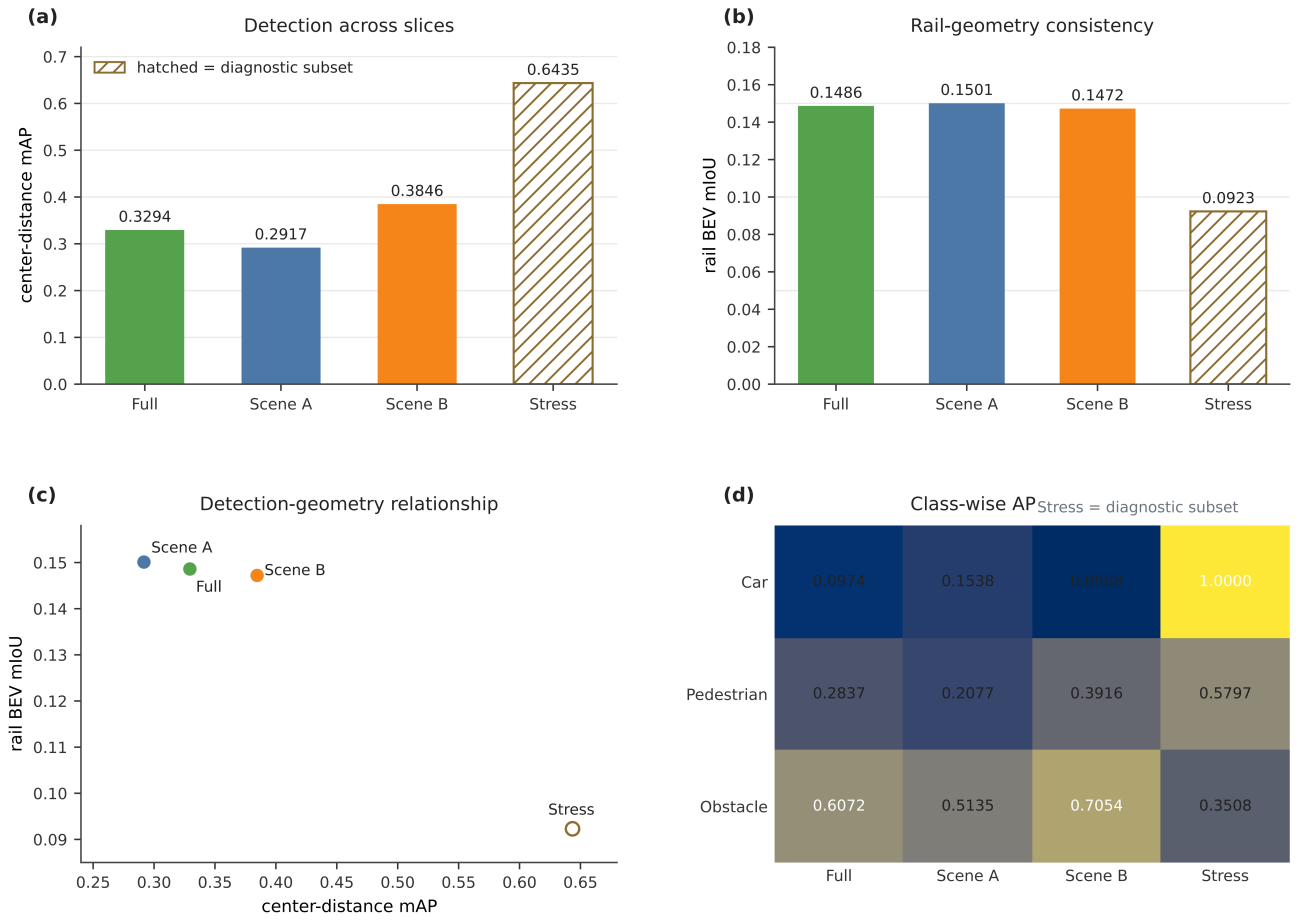
Scene-level and operating-domain variation of Rail-BEV. The diagnostic slices are used to interpret domain sensitivity and should not be treated as replacements for the independent test benchmark.

**Figure 6:**
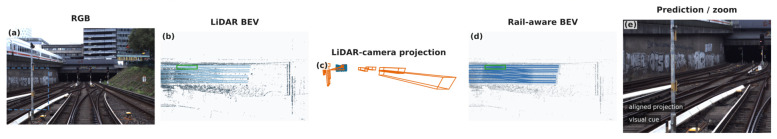
Multimodal cross-modal alignment and diagnostic analysis of the proposed Rail-BEV framework. (**a**) The front-center RGB image; (**b**) LiDAR BEV representation with a highlighted region of interest in a green frame; (**c**) LiDAR-camera projection showing the geometric alignment of objects; (**d**) Rail-aware BEV interpretation, where the corresponding rail structure is highlighted in the green frame; (**e**) Prediction and zoomed diagnostic view demonstrating the aligned projection and visual cues. Specifically, we select an intuitive example of cross-modal alignment: the green frame in (**b**) and the blue volumetric part in (**c**) undergo cross-modal alignment, and their correct spatial alignment is successfully verified in (**d**,**e**).

Figure 5 provides a representative qualitative visualization of the multimodal sensing pathway in a curved railway scene. The figure follows the evidence chain from the front-center RGB image and LiDAR BEV representation to LiDAR-camera projection, rail-aware BEV interpretation, and a zoomed diagnostic view. This visualization complements the quantitative results by showing how lightweight visual evidence is geometrically associated with LiDAR structure before being organized in the BEV space, see Figure 6.

### 4.6. Supplementary Railway-Oriented Localization Diagnostics

Although center-distance AP/mAP and rail BEV mIoU are the main reported metrics, strict railway-oriented localization diagnostics are useful for identifying remaining safety-relevant bottlenecks. Table 4 reports RA-AP and RA-mAP under strict aligned evaluation and oracle-center diagnosis. The strict aligned evaluation obtains 0.1865 obstacle RA-AP and 0.0747 overall RA-mAP. The oracle-center diagnostic yields 0.0853 overall RA-mAP, indicating that improved center handling alone does not fully resolve strict railway-oriented localization.

These diagnostic values should not be promoted to the primary benchmark headline. Instead, they reveal that strict rail-corridor localization remains more challenging than the center-distance benchmark suggests. This is expected in long-range railway perception, where sparse far-field returns and small lateral deviations can substantially affect corridor-intrusion interpretation.

The conceptual distinction between center-distance matching and stricter railway-oriented localization diagnostics is further illustrated in Figure S3.

### 4.7. Summary of Results

Overall, the revised results support three main findings: First, the distance-stratified baseline comparison shows that Rail-BEV achieves the strongest overall mAP among the compared methods and provides a marked improvement for long-range pedestrian detection. Second, the controlled ablation study shows that front-view RGB assistance improves the LiDAR-only baseline from 0.5612 to 0.5750 mAP, while ROI-based rail-corridor refinement further increases mAP to 0.5916 and rail BEV mIoU to 0.1193. Third, the additional track-consistency refinement does not provide a monotonic gain under the current fixed protocol, indicating that ROI-based structural refinement is the more stable component in the present framework.

**Figure 7:**
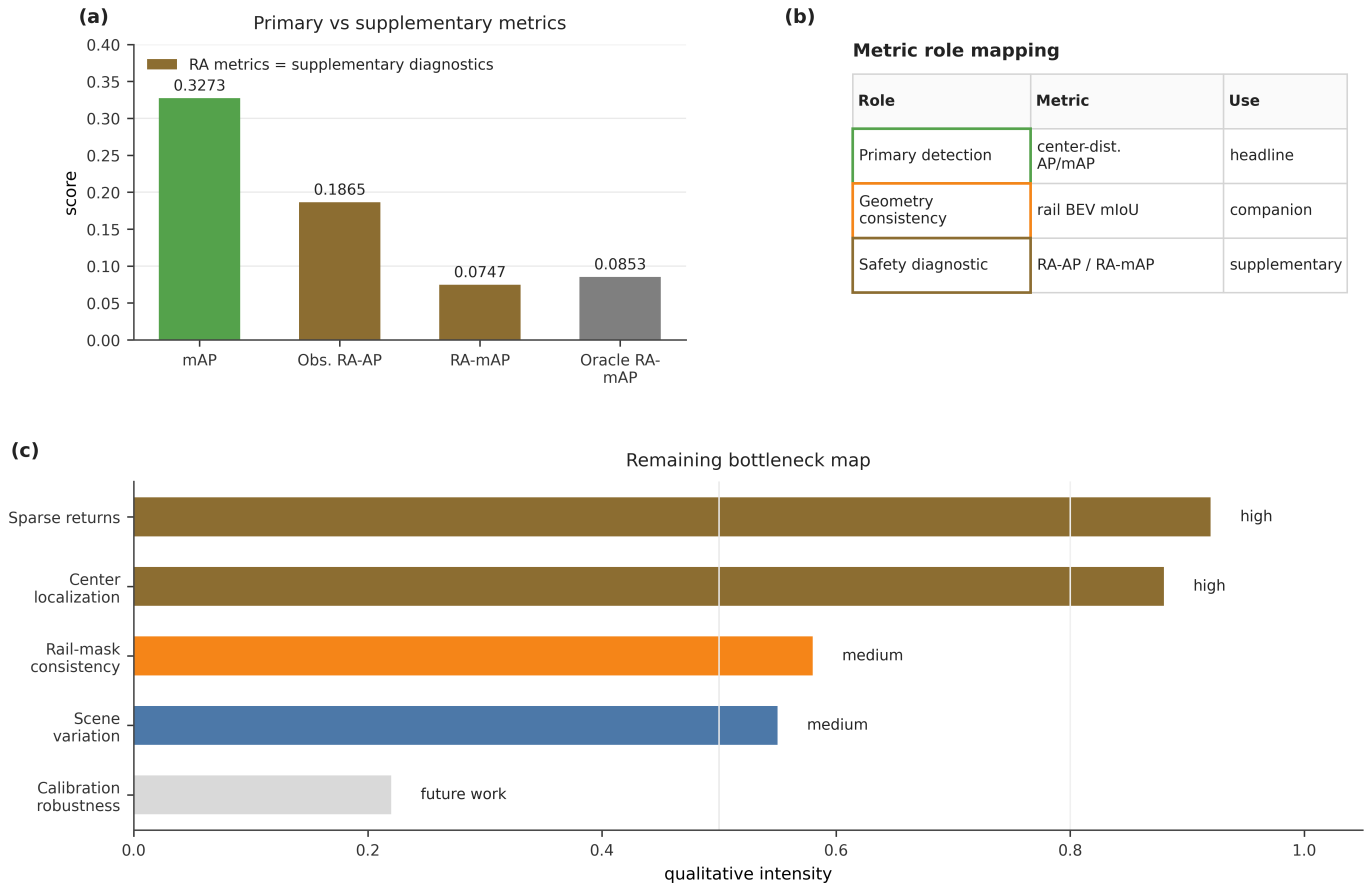
Performance metrics and diagnostic bottleneck analysis. (**a**) Comparison of primary and supplementary evaluation metrics; (**b**) Mapping of metric roles to their respective usage in the evaluation framework; (**c**) Qualitative intensity of remaining bottlenecks. The color coding in the figures consistently represents the functional role of each metric: green denotes primary detection, orange/brown indicates geometry consistency or high-intensity bottlenecks, blue signifies scene-related variations, and grey represents future evaluation aspects.

At the same time, the results define a clear scope for interpretation. The scene-isolated public benchmark reduces temporal leakage relative to random frame sampling, but it remains limited by the scale and diversity of available public railway sequences. The most defensible interpretation is therefore that Rail-BEV provides an initial reproducible LiDAR-centric, front-view-assisted, rail-aware BEV baseline for long-range railway obstacle perception, while broader operating-domain coverage and stricter localization robustness remain important future directions, see Figure 7.

## 5. Discussion

### 5.1. Sensor-Hierarchy Interpretation

The results support the central design premise of Rail-BEV: long-range railway obstacle perception benefits from a LiDAR-centric sensing hierarchy rather than from treating all modalities as equally weighted streams. LiDAR remains the primary source of metric geometry, which is essential for reasoning about obstacle position in a structured rail corridor [1,2,3,12,23,26,27]. The front-center RGB camera contributes as a lightweight auxiliary sensor by providing appearance cues that are geometrically associated with LiDAR through calibrated projection before being organized in the BEV space [9,13,14]. This configuration is particularly suitable for railway operation, where the safety-critical field of view is concentrated along the forward rail corridor, and where full surround-view camera fusion would introduce additional calibration and runtime complexity.

The revised sensor-configuration results support this interpretation. Under the unified scene-isolated evaluation setting, the LiDAR-only baseline reaches 0.5612 mAP and 0.0533 rail BEV mIoU, whereas adding the front-center RGB stream increases the corresponding values to 0.5750 and 0.0583, respectively. The improvement is especially meaningful because the visual stream is not used as a heavy independent perception branch; it is introduced as aligned auxiliary evidence within a LiDAR-centered BEV backend. Therefore, the gain should be interpreted as evidence for a practical sensor-organization strategy: forward visual assistance can strengthen sparse long-range LiDAR perception when it is constrained by calibrated geometry and integrated in a common BEV representation.

The distance-stratified comparison further indicates that the advantage of Rail-BEV is most visible in long-range pedestrian perception. In the 100+ m band, Rail-BEV achieves 36.81% pedestrian AP, whereas the corresponding values for CenterPoint and BEVFusion are 2.86% and 0.34%, respectively. This result should not be over-interpreted as full operational-domain generalization; rather, it shows that the proposed LiDAR-centered, front-view-assisted, rail-aware design can improve reproducible long-range perception under the current scene-isolated public benchmark.

### 5.2. Effect of Rail-Aware Structural Reasoning

The rail-aware branch and inference-time refinement further indicate that railway perception should not be evaluated solely as generic object detection in an unconstrained BEV plane. In railway scenes, the operational meaning of a detection is strongly conditioned by its relationship to the rail corridor and clearance envelope [24,25]. The controlled ablation shows that ROI-based rail-corridor refinement increases the overall mAP from 0.5750 to 0.5916 and improves the rail BEV mIoU from 0.0583 to 0.1193. This supports the interpretation that coarse corridor-level geometry helps organize railway-relevant BEV evidence.

At the same time, the structural-refinement results should be interpreted with caution. The improvement is not uniformly positive across all object categories: obstacle AP decreases from 0.3398 to 0.3109 after ROI refinement, while pedestrian AP also decreases slightly from 0.4349 to 0.4242. The additional track-consistency refinement reaches 0.5793 mAP and 0.0665 rail BEV mIoU, which is lower than the ROI-only variant. Thus, the revised evidence supports ROI refinement as the more stable structural component, while TC remains a supplementary refinement variant requiring further validation.

The rail BEV mIoU results also require balanced interpretation. The ROI-refined value of 0.1193 is useful as a companion geometry-consistency indicator, but it should not be presented as evidence of high-quality dense rail segmentation. In this work, the rail branch primarily serves as a coarse corridor-level structural cue for BEV perception and as a means of evaluating rail-geometry consistency. Its value lies in improving the organization of railway-relevant evidence, rather than in replacing specialized rail parsing or track reconstruction systems.

### 5.3. Operating-Domain Variation and Metric Scope

The scene-level analysis shows that the benchmark behavior is sensitive to operating-domain composition. Although the revised split separates training, validation, and testing at the sequence level, the public test sequences remain biased toward specific station and signal scenes. Therefore, the results should be interpreted as reproducible evidence under a controlled cross-scene public split, rather than as a final validation across the full railway operational design domain.

The operating-domain diagnostics further illustrate why selected slices should not be treated as global benchmark replacements. Detection AP and rail BEV mIoU quantify different aspects of performance: a model may approximately localize object centers while still producing coarse or fragmented rail masks. This discrepancy justifies the use of rail BEV mIoU as a companion geometry-consistency metric alongside center-distance mAP, rather than as the sole evidence for rail-geometry quality.

The supplementary railway-oriented localization diagnostics reveal an additional limitation. Under strict aligned evaluation, the obstacle RA-AP is 0.1865 and the overall RA-mAP is 0.0747. The oracle-center diagnostic slightly increases the overall RA-mAP to 0.0853 but does not consistently improve obstacle-specific RA-AP. This pattern suggests that strict railway-oriented localization is affected by more than center placement alone [11]. Box geometry, lateral alignment, rail-corridor relation, and sparse far-field returns all contribute to the remaining gap between center-distance detection performance and strict safety-oriented localization behavior.

### 5.4. Limitations

The first limitation is the scope of the scene-isolated public benchmark. Although the revised protocol uses 23 training sequences, 5 validation sequences, and 5 independent test sequences, the available public data remain limited relative to the diversity of real railway operating domains [12]. Consequently, the results should be presented as evidence for a controlled LiDAR-centric railway sensing baseline, rather than as a final generalization claim across severe curves, tunnels, open mainline sections, night scenes, active adverse weather, or rare operational anomalies [26,27,30,31].

The second limitation concerns multimodal robustness. The proposed configuration intentionally retains only the front-center RGB camera as auxiliary visual evidence. This choice is aligned with forward-looking railway safety perception and reduces sensing complexity, but it does not exhaust the full design space of multi-view camera assistance, radar complementarity, adverse-weather sensing, or redundancy under sensor degradation [29]. Future evaluations should therefore include controlled sensor-dropout tests, calibration perturbation studies, and broader weather and illumination conditions to determine how robust the frontal-clean design remains outside nominal scenarios [16,32].

The third limitation is that temporal and ego-motion effects are not used as headline evidence in the present paper. Claims about multi-frame fusion, GNSS/IMU-supported motion compensation, or temporal consistency should therefore be reserved for future studies unless they are supported by unified reruns under the same evaluation protocol.

### 5.5. Deployment-Oriented Efficiency Considerations

Although Rail-BEV is not claimed as a fully optimized real-time onboard system, the current design follows a deployment-conscious sensing hierarchy. The framework avoids full surround-view camera fusion and retains only the front-center RGB camera as auxiliary visual evidence, thereby limiting the multimodal interface to the safety-critical forward rail corridor [1,2,3,12,31,33]. This design reduces cross-view calibration complexity and keeps LiDAR as the dominant geometric backbone. The present results should therefore be interpreted as a compact sensor-aware railway perception baseline rather than as a computationally minimized embedded product. Future onboard implementation should report platform-specific latency, memory footprint, calibration maintenance cost, and failure behavior under sensor degradation before deployment-level claims are made.

### 5.6. Future Directions

Future work should first extend the evaluation to broader scene coverage and more diverse operating domains, including curved tracks, tunnels, open mainline sections, night scenes, adverse weather, and stations with different platform layouts [26,27,30,31]. A larger and more diverse evaluation protocol would make it possible to separate model behavior caused by the sensing architecture from behavior caused by scene composition. Second, calibration robustness should be tested explicitly because the proposed RGB–LiDAR fusion relies on LiDAR-to-image projection. Even small projection errors may reduce the benefit of front-view visual assistance or weaken rail-aware refinement [16,32]. A further direction is to unify the main center-distance benchmark with stricter railway-oriented localization diagnostics. Finally, temporal and ego-motion modeling should be revisited under a controlled experimental design so that any claimed benefit can be separated from checkpoint selection, structural refinement, and sensor-configuration effects.

## 6. Conclusions

This study presents Rail-BEV as a LiDAR-centric and sensor-aware bird’s-eye-view (BEV) perception framework for long-range railway obstacle detection. The framework retains LiDAR as the primary geometric sensing modality and uses only the front-center RGB camera as lightweight auxiliary visual evidence through calibrated LiDAR-to-image projection [12,13,14]. By organizing the aligned geometric and visual cues within a railway-oriented BEV backend, Rail-BEV integrates geometry-aware fusion, rail-geometry prediction, and lightweight structural refinement into a compact sensing pipeline tailored to the forward rail corridor.

Under a unified scene-isolated railway benchmark, Rail-BEV achieves the strongest overall mAP among the compared methods, reaching 0.6669 and showing a particularly large improvement for long-range pedestrian perception. The controlled ablation further shows that front-view RGB assistance improves the LiDAR-only baseline from 0.5612 to 0.5750 mAP, while ROI-based rail-corridor refinement further increases the overall mAP to 0.5916 and rail BEV mIoU to 0.1193. The additional track-consistency refinement produces limited incremental benefit under the current setting, indicating that ROI-based structural refinement is the more reliable component in the present framework.

These findings suggest that railway obstacle perception benefits from a LiDAR-centered sensing hierarchy supplemented by lightweight visual and structural cues. At the same time, the results should be interpreted within clear boundaries. The rail-geometry branch should be regarded as a coarse corridor prior rather than as a high-fidelity rail reconstruction module, and strict railway-oriented localization remains more difficult than center-distance matching. Future work should extend the evaluation to more diverse railway scenes, investigate calibration drift and sensor-degradation robustness, and assess real-time performance on onboard railway computing platforms.

## Figures and Tables

**Table 1:** Distance-stratified performance comparison (AP,%) under the scene-isolated OSDaR23 evaluation setting.

Method	Class	0–50 m	50–100 m	100+ m	Overall AP	mAP
BEVFusion	Car	99.00	98.00	N/A	98.50	50.18
	Pedestrian	43.63	4.03	0.34	35.57	
	Obstacle	0.00	24.90	0.00	16.46	
CenterPoint official	Car	99.00	99.00	N/A	99.00	52.24
	Pedestrian	46.89	11.47	2.86	39.62	
	Obstacle	0.00	29.47	0.00	18.11	
**Rail-BEV (ours)**	Car	**100.00**	**99.94**	N/A	**99.96**	**66.69**
	Pedestrian	**66.16**	**46.92**	**36.81**	**62.23**	
	Obstacle	**41.28**	22.84	**0.13**	**37.90**	

Notes: Bold values indicate the best performance. All methods were evaluated under the same scene-level split, distance bands, class definitions, and center-distance AP calculation.

**Table 2:** Clean ablation results under the unified scene-isolated evaluation setting.

Variant	Car AP	Pedestrian AP	Obstacle AP	mAP	Rail BEV mIoU	Interpretation
Base (LiDAR-only)	1.0000	0.4565	0.2270	0.5612	0.0533	Geometric baseline
Base + RGB	1.0000	0.4349	0.3398	0.5750	0.0583	Front-view visual assistance
Base + RGB + ROI refinement	0.9900	0.4242	0.3109	0.5916	0.1193	Most stable structural gain
Base + RGB + ROI + TC	1.0000	0.4062	0.3317	0.5793	0.0665	Limited incremental gain

Notes: ROI denotes rail-corridor region-of-interest refinement, and TC denotes track-consistency refinement. All variants were evaluated once on independent test sequences using the final training checkpoint; no validation-selected or retained-strongest checkpoint was used.

**Table 3:** Scene-level data partition and evaluation scope.

Split	Seq.	Frames	Use	Scene-Level Split	Role	Notes
Training	23	583	Model fitting	Yes	Learning	No test frames
Validation	5	50	Hyperparameter check	Yes	Development	Not final reporting
Test	5	230	Independent evaluation	Yes	Final reporting	Held out
**Total**	**33**	**863**	**Public sequence benchmark**	**Yes**	**Controlled scope**	**Not full ODD validation**

Notes: Bold text indicates the overall summary of the dataset partitions. The test split is scene-isolated and is used only for final reporting. Broader operational-domain validation remains necessary for future work.

**Table 4:** Quantitative results of strict railway-oriented localization diagnostics.

Diagnostic Setup	Obstacle RA-AP	Overall RA-mAP	Interpretation
Strict aligned evaluation	0.1865	0.0747	Current strict railway-oriented localization bottleneck
Oracle-center diagnostic	0.0591	0.0853	Improved center handling still leaves room for localization improvement

Notes: RA-AP and RA-mAP are retained as supplementary diagnostics only. They are not used as the primary benchmark metrics.

## Data Availability

Publicly available datasets were analyzed in this study. The OSDaR23 dataset can be found here: https://data.fid-move.de/dataset/osdar23, accessed on 20 May 2026.

## Supplementary Materials

The following supporting information can be downloaded at https://www.mdpi.com/article/10.3390/s26123637/s1.
