# Supplementary material for: Rail-BEV: A LiDAR-Centric and Sensor-Aware BEV Perception Framework for Long-Range Railway Obstacle Detection
Source: Sensors (Basel). 2026 Jun 7;26(12):3637. doi: 10.3390/s26123637 (PMC13306471; doi:10.3390/s26123637)
Supplement: Supplementary file 1 [file sensors-26-03637-s001.zip › sensors-4325085-supplementary.pdf]

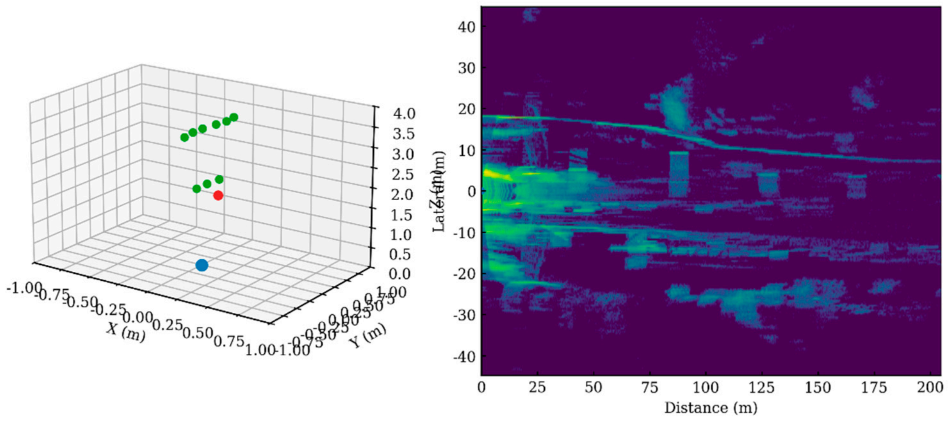

Fig. S1. Sensor Layout and Effective Field-of-View (FoV) Coverage.

Text.S1.

Schematic of the multi-sensor configuration and fused point cloud coverage. The left panel illustrates the relative poses of the sensors after calibration. The right panel displays the empirical coverage density in the Bird's-Eye View (BEV), demonstrating continuous sensing coverage along the railway corridor and physically interpretable sparsity in the extreme long-range areas.

Table S1. Sensor-configuration comparison under the frontal-clean aligned protocol.

**Configuration**	**car AP**	**pedestrian AP**	**obstacle AP**	**mAP**	**rail BEV mIoU**	**Interpretation**
**LiDAR-only aligned, best checkpoint**	**0.1343**	**0.0662**	**0.2664**	**0.1556**	**0.046**	**Geometric-only control**
**LiDAR + front-center aligned base**	**0.0652**	**0.1866**	**0.4377**	**0.2298**	**0.1412**	**Sensor-aware base**
**Base + C + D, best checkpoint**	**0.0974**	**0.2774**	**0.607**	**0.3273**	**0.1486**	**Best-checkpoint rerun**
**Base + C + D, archived strongest**	**0.0974**	**0.2837**	**0.6072**	**0.3294**	**0.1486**	**Retained strongest line**

Text.S2.

Table S1 provides the clearest sensor-level evidence in the current study. Under the same repository-defined frontal-clean aligned protocol, the LiDAR-only baseline reaches `0.1556` mAP and `0.0460` rail BEV mIoU, while the aligned LiDAR-plus-front-center baseline reaches `0.2298` mAP and `0.1412` rail BEV mIoU. This result directly supports the main manuscript claim that lightweight front-view RGB assistance improves the present LiDAR-centered railway sensing pipeline when the two modalities are organized through calibrated alignment and a unified BEV representation.

The strongest practical line is obtained when the aligned visual configuration is combined with rail-aware structural refinement. Using the best checkpoint , the `Base + C + D` pipeline reaches `0.3273` mAP and `0.1486` rail BEV mIoU, which is already very close to the retained strongest archived line of `0.3294` mAP.

Table S2. Deployment-oriented structural refinement ablation.

Configuration	car AP	pedestrian AP	obstacle AP	mAP	rail BEV mIoU	Interpretation
Base	0.0652	0.1866	0.4377	0.2298	0.1412	Aligned visual base
Base + C	0.0652	0.2079	0.5396	0.2709	0.1412	ROI geometry refine gain
Base + C + D, latest	0.0671	0.2079	0.5396	0.2715	0.1412	Latest-checkpoint rerun
Base + C + D, best checkpoint	0.0974	0.2774	0.607	0.3273	0.1486	Best-checkpoint rerun

Text.S3.

Table S2 isolates the contribution of rail-aware structural refinement. Starting from the aligned visual base, adding `C` raises mAP from `0.2298` to `0.2709` and obstacle AP from `0.4377` to `0.5396`. This indicates that ROI geometry refine is the clearest isolated source of gain among the current deployment-oriented modules. It improves the detector by enforcing corridor-consistent structural reasoning rather than by adding a new sensor branch.

Adding `D` on top of `Base + C` requires careful interpretation. The latest-checkpoint rerun reaches `0.2715` mAP, whereas the best-checkpoint rerun rises to `0.3273` mAP. For this reason, the supplementary material explicitly distinguishes `latest-checkpoint rerun`, `best-checkpoint rerun`, and the retained archived strongest result, preventing the final pipeline from being presented as a single ambiguous benchmark line.

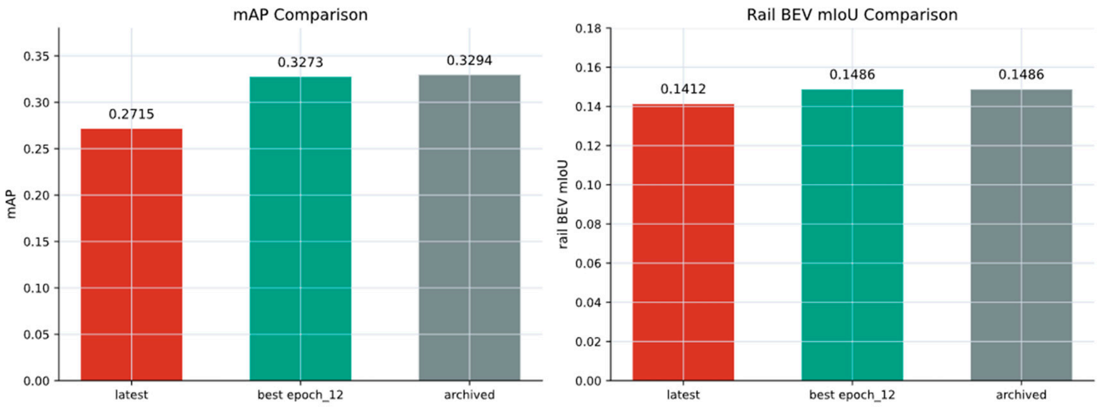

Figure S2. Checkpoint-selection effect on the final Base + C + D pipeline.

Text.S4.

The figure compares the latest-checkpoint rerun, the best-checkpoint rerun, and the historical archived strongest result for the final rail-aware pipeline. It illustrates that `latest.pth` should not be interpreted as the strongest available checkpoint and that the best-checkpoint rerun is already very close to the retained archived best.

Table S3. Scene-level and operating-domain slices.

Slice	car AP	pedestrian AP	obstacle AP	mAP	rail BEV mIoU	Use
**Full validation**	**0.0974**	**0.2837**	**0.6072**	**0.3294**	**0.1486**	**Primary benchmark**
**Scene 4.3**	**0.1538**	**0.2077**	**0.5135**	**0.2917**	**0.1501**	**Nominal slice**
**Scene 4.4**	**0.0568**	**0.3916**	**0.7054**	**0.3846**	**0.1472**	**Nominal slice**
**Far-range stress**	**1**	**0.5797**	**0.3508**	**0.6435**	**0.0923**	**Diagnostic slice**

**subset**

Text.S5.

Table S3 is used to describe operating-domain variation rather than to replace the primary benchmark. The two nominal station slices already reveal class-wise differences under the same aligned protocol. Meanwhile, the far-range stress subset highlights that a high filtered mAP can coexist with weak rail-geometry consistency. This observation explains why rail BEV mIoU is retained as a companion structural metric and why the stress slice is interpreted as diagnostic evidence rather than as the headline benchmark.

Figure S3. Conceptual comparison between center-distance matching and strict railway-oriented localization diagnostics.

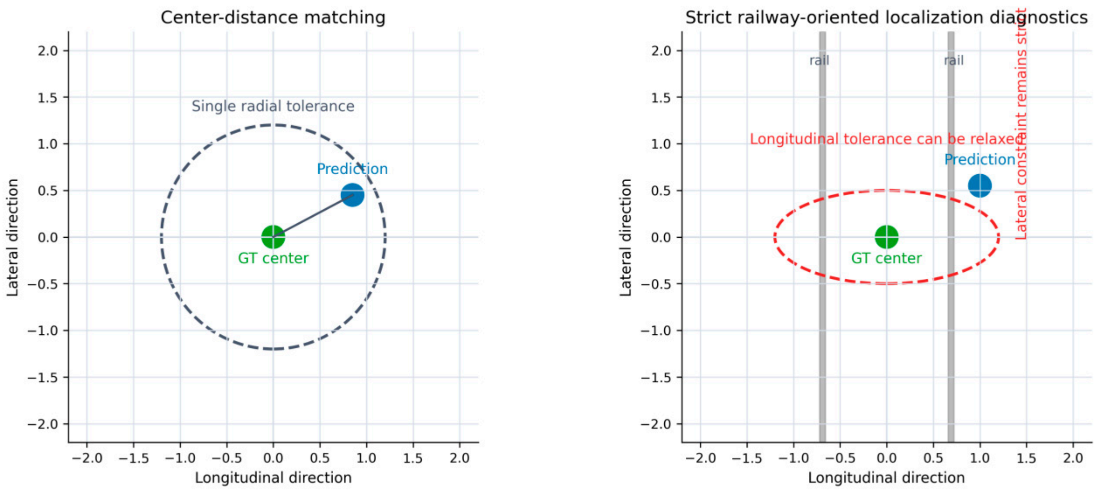

Text.S6.

The figure contrasts the primary benchmark metric used in the main manuscript with a stricter railway-oriented localization interpretation. It is intended to clarify why center-distance AP/mAP and RA-oriented diagnostics capture different aspects of performance and should not be treated as interchangeable.

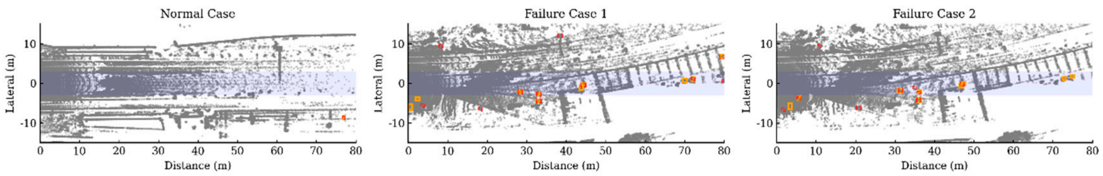

Fig.S4. Visualization and Analysis of Failure Cases Under Extreme Conditions.

Text.S7.

Illustration of typical failure mechanisms categorized by physical, algorithmic, and temporal levels. These cases reveal the physical limits of the perception system under extreme meteorological conditions (e.g., severe point cloud attenuation) and complex geometric topologies (e.g., track mask derivation errors).

Failure Mode	Quantitative Definition	Count	Proportion
**Physical Limit**	Recall ≤ 0.05 & FN ≥ 2 & Obstacle FN ≥ 1	2	1.37%
**Algorithm Degradation**	Precision ≤ 0.60 & FP ≥ 1 & Recall > 0.05	7	4.79%
**Temporal Disruption**	Consecutive (≥ 2) frames with Recall ≤ 0.05 & FN ≥ 1	60	41.10%

Table S4. Quantitative Analysis of Failure Modes under Extreme Conditions

Text.S8.

The quantitative failure analysis was evaluated on a carefully filtered subset of 146 high-difficulty extreme scenario frames from the OSDaR23 test set. As indicated by the statistical distribution, absolute physical feature loss (Physical Limit) and track mask derivation errors (Algorithm Degradation) only account for a marginal fraction of failures. The dominant failure mechanism is Temporal Disruption (41.10%), which occurs when the spatiotemporal manifold module loses historical feature support due to severe occlusion or continuous point cloud attenuation.

Figure S5. Detection accuracy versus GPU memory trade-off.Text.S6.

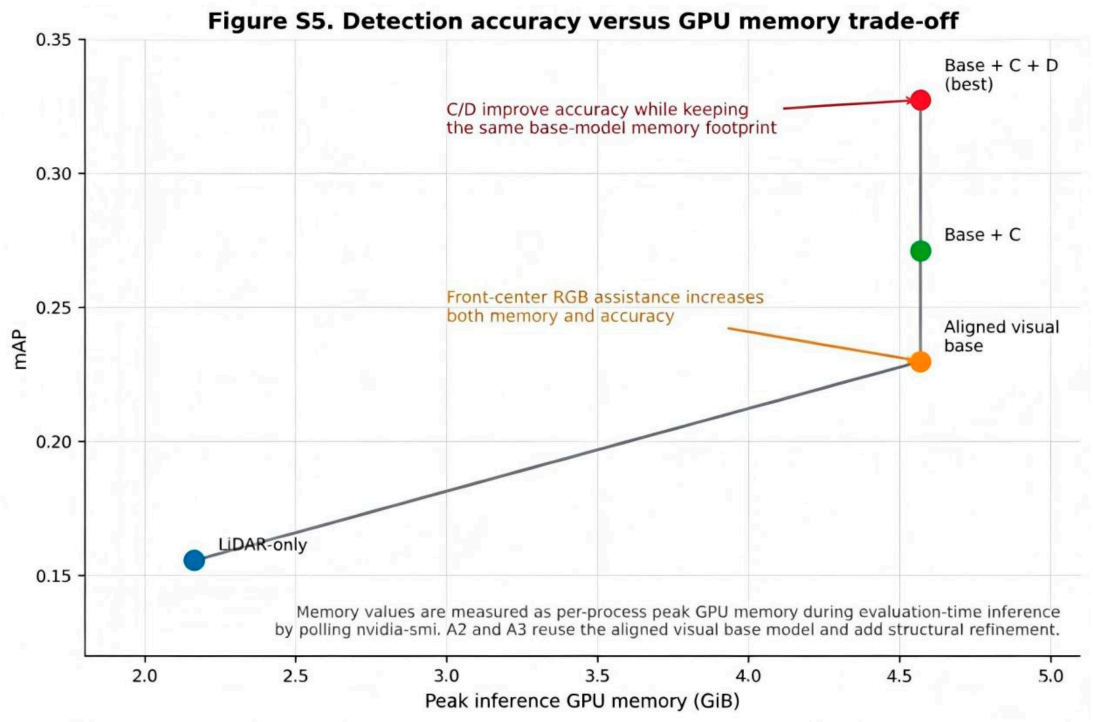

Configuration	Peak inference GPU memory (GiB)	mAP	Source note
**LiDAR-only aligned, best checkpoint**	**2.162**	**0.1556**	**per-process nvidia-smi peak during evaluation**
**Aligned visual base**	**4.57**	**0.2298**	**per-process nvidia-smi peak during evaluation**
**Base + C**	**4.57**	**0.2709**	**per-process nvidia-smi peak during evaluation**
**Base + C + D, best checkpoint**	**4.57**	**0.3273**	**per-process nvidia-smi peak during evaluation**

Table S5. Accuracy-memory summary for the current main configurations.

Text.S9.

Figure S5 and Table S5 provide an optional deployment-oriented view of the present system. Introducing front-center RGB assistance increases the peak inference GPU memory footprint from about `2.162` GiB to `4.570` GiB, while also improving mAP from `0.1556` to `0.2298`. After that, `C` and `D` improve detection accuracy without introducing a new backbone-level memory regime in the current repository, because they operate as structural

additions on the aligned visual base.

The reported memory values are measured as per-process peak GPU memory during evaluation-time inference by polling `nvidia-smi` throughout each repository-aligned rerun. They should therefore be interpreted as inference-side peak GPU memory for the evaluated process chain rather than as training memory or as a synthetic analytical estimate.
